# Improving Alzheimer's Disease Detection for Speech Based on Feature Purification Network

**DOI:** 10.3389/fpubh.2021.835960

**Published:** 2022-03-03

**Authors:** Ning Liu, Zhenming Yuan, Qingfeng Tang

**Affiliations:** ^1^School of Public Health, Hangzhou Normal University, Hangzhou, China; ^2^Department of Mathematics and Computer Science, Quanzhou Normal University, Quanzhou, China; ^3^Fujian Provincial Key Laboratory of Data-Intensive Computing, Quanzhou Normal University, Quanzhou, China; ^4^School of Information Science and Technology, Hangzhou Normal University, Hangzhou, China; ^5^School of Computer and Information, Anqing Normal University, Anqing, China

**Keywords:** Alzheimer's disease, natural language processing, deep learning, transformer, machine learning, speech and language, mild cognitive impairment

## Abstract

Alzheimer's disease (AD) is a neurodegenerative disease involving the decline of cognitive ability with illness progresses. At present, the diagnosis of AD mainly depends on the interviews between patients and doctors, which is slow, expensive, and subjective, so it is not a better solution to recognize AD using the currently available neuropsychological examinations and clinical diagnostic criteria. A recent study has indicated the potential of language analysis for AD diagnosis. In this study, we proposed a novel feature purification network that can improve the representation learning of transformer model further. Though transformer has made great progress in generating discriminative features because of its long-distance reasoning ability, there is still room for improvement. There exist many common features that are not indicative of any specific class, and we rule out the influence of common features from traditional features extracted by transformer encoder and can get more discriminative features for classification. We apply this method to improve transformer's performance on three public dementia datasets and get improved classification results markedly. Specifically, the method on Pitt datasets gets state-of-the-art (SOTA) result.

## Introduction

Alzheimer's disease (AD) is a nervous degenerative disease with an insidious and irreversible onset, which is difficult to be detected in every stage. AD can influence patients' daily living ability and social communicate ability and may even lead to disability (1, 2). Researchers have found that AD has a profound impact on patients' language function (3) in addition to mood, attention, memory, movement, and so on. Language is the representation of mental activities, which can clearly reflect the relationship among language, cognition, and communication (4). Language interference is a common manifestation of patients (5) with AD which may even earlier than orientation and memory difficulties (6, 7). Picture description task, taken from Boston Aphasia Diagnostic Test (8), has already been verified sensitive to subtle cognitive deficits (9); therefore, valuable clinical information can be obtained from spontaneous speech to recognize AD. The transcripts of speech can be used to detect AD effectively.

The problem of AD recognition can be regarded as text classification problem in natural language processing (NLP). Deep learning models manifest better in classification as they can extract deep semantic features by effective model architecture automatically. For example, RNN can capture long-term dependencies within sentence, but it may neglect some important local words which may important for classification (10), and CNN can capture local and position-related features (11) but cannot give enough weight to some discriminative or special words. To solve the problem, attention mechanism was introduced. Transformer gives different weights to different words using attention mechanism, the performance of which is better than CNN and RNN. Although transformer has made great progress in producing discriminative features by powerful representation learning, there is still room to improve. There are few studies nowadays in this area to improve representation learning of deep learning. Based on GRL (12–17) in extracting common features which are not discriminative for classification, this paper proposes a novel feature purification method to improve the representation learning of transformer to get a more discriminative feature vector to diagnose AD.

The original transcripts of speech are the description of a picture, which should be comprehensive and integrated for a normal individual. That is to say, the discriminative words or sentences, with relevant and less vague words, should be included. For example, accurate descriptive words, such as “mother”, “tap”, “the stool is tipping”, etc., are usually a better cognitive sign. Words or sentences such as “I do not know”, “um”, and “pause” should be an indicative of a bad cognitive condition, and they are discriminative for AD recognition. But some equivocal, inconsequential, and even irrelevant descriptions are unhelpful and may even interfere with the final classification, such as “is not that enough?”, “It is great”, “there may be a little breeze coming in”, et al. They can disturb the representation learning of deep learning by producing suboptimal representations. To solve this problem, transformer proposes a self-attention mechanism to give weight to words and usually can get better performance than RNN and CNN. Though attention may alleviate the influence by giving a higher or lower weight for those more or less relevant words, the classification problem cannot be solved properly with inaccurate attention mechanism or specificity of data. To solve the above problems, our study, inspired by the paper (17) which used feature projection method to purify the representation learning of deep learning, proposes a novel feature purification method to improve representation learning of transformer to get more discriminative features, which is GP-Net. It has two subnetworks, a common feature learning network called G-Net and a purification network called P-Net. G-Net uses gradient reverse layer (GRL) (12, 13, 18) to extract common features which are shared by classes and have no or few roles for classification. P-Net first uses transformer encoder to extract traditional feature vector for the sentence. Then, it rules out the common features from traditional feature vector to generate more purified features. It is clear that this operation gets rid of the effect of common features and makes the system only focus on discriminative features. We will explain the principle in Method Section.

The experiments on three datasets with our method get an improved performance which prove that the purified features are more discriminative. To the best of our knowledge, there have been still no studies to recognize AD from spontaneous speech by purifying representation learning of deep learning up to now.

The key contributions we have made in this work include the following:

(1) A whole process of AD screening method, based on linguistic data, was designed and implemented.
(2) We propose a novel feature purification network to improve representation learning of transformer and get state-of-the-art (SOTA) result on Pitt dataset.
(3) The proposed method has the advantage of low cost, reliable, and convenience, which can provide a feasible solution for the screening of AD with a better performance.

## Related Work

Existing studies on AD diagnosis across spontaneous speech mainly focus on two aspects. One is feature extraction manually including acoustic features (19–21), linguistic features (22–25), or their combinations (21). This method is subjective and needs more professional knowledge. They are generally associated with a specific task scenario; once the scenario changes, these artificially designed features and prior settings cannot adapt to new scenarios and need to be redesigned, so the model has a low universality. The other is deep learning method which can extract deep semantic features automatically. Based on its powerful representation learning ability, the performance of deep learning is usually better than the first method. Additionally, deep learning improves the generalization ability of the classifiers which can be utilized further in different clinical environments. Deep neural network can process representation learning to extract deep semantic features using cascaded data of multilevel non-linear processing units without the need for feature engineering manually.

### AD Detection Based on Deep Learning

There are many studies to detect AD from oral speech with deep learning methods (26–29), such as RNN, long–short-term memory (LSTM) networks [e.g., ELMo (30)] and CNN. Recurrent convolutional neural Networks (RCNN) (31) uses Bi-LSTM to get contextual information and then concedes the hidden output of Bi-LSTM and word embedding for classification. DPCNN (32) is a simple network with 15 layers which likes a deep CNN, and it increases network depth of CNN but does not increase the computational cost. Attention mechanism is used in many NLP tasks such as text classification (33–36). Transformer architecture [e.g., Bert (37)] uses attention mechanism to extract deep semantic features. Enhanced representation through knowledge integration (ERNIE) (38, 39), proposed in 2019 by Baidu corporation, is optimized further based on Bert model. They usually have better performance than CNN, RNN, and LSTM.

Public Dementiabank datasets or ADReSS challenge (40) datasets are often used to recognize AD. For example, Orimaye et al. (41) proposed the combination of deep language models and deep neural network to predict mild cognitive impairment (MCI) and AD. The datasets used were public Dementiabank transcript with 37 healthy elderly and 37 MCI transcripts. The study did not use any handcrafted features; just the original transcripts were fed to the model, and n-gram word embedding method combined with deep neural network (DNN) got a best AUC of 0.83. Different from our dataset and classification, there was no comparability with our method. Karlekar et al. (23) used four types of interviews: story recall, sentence construction, cookie-theft picture description, and vocabulary fluency; the dataset included 243 normal controls and 1,017 AD transcripts. Three classifiers were used for comparison, that is, LSTM-RNN, CNN, and CNN-LSTM, and achieved a best accuracy of 91.1%, but the results were somewhat questionable as mentioned in the Discussion. These methods used deep learning algorithms or their linear combinations to recognize MCI and AD. Our work is much different clearly as none of these existing studies improve representation learning of deep learning by feature purification method.

### Studies Related to GRL

Our study is related to some former work. Ganin and Lempitsky (13) first introduced GRL to extract common features which were sentiment-sensitive and domain shared in domain adaptation (DA). It embeds DA to the process of representation learning in order that the final classification result is more discriminative for the domain changes. Though we use GRL to extract common features, we do not use it in the area of DA, and they also do not use for feature purification. Belinkov et al. (14) used adversarial learning to encourage the model to process representation learning on SNLI dataset. Combining with aspect attention and GRL, Kai Zhang et al. (16) studied cross domain text classification problem, and common features across domains were extracted from the aspects for text classifications. The idea of generative adversarial networks (GANs) (42) was used to ensure that the common feature space did not mix with private features and only contained pure task-independent common feature representation. In these studies, they all used GRL to extract common features inseparable for two domains, and domain-shared features were generated in the shared space according to adversarial training, whereas our study is different from them clearly as this existing work does not improve representation learning of the model. The study (17) proposed a feature projection method to further improve representation learning of deep learning from a novel angle. The method projected existing features into the orthogonal space of the common features, so the resulting projection is perpendicular to the space that common features located in and thus more discriminative for text classification. Different from this study (17) which only deletes a section of common features, we rule out the influence of whole common features, which we believe that a better classification performance should be achieved. Also, we did the experiment with the method of study (17) on Pitt dataset, and the performance is not better than our method, just as shown in Table 1.

**Table 1:** Relationship between predicted and true classes.

	**True class**	
**Predicted class**	Positive	Negative
Positive	True positive (TP)	False positive (FP)
Negative	False negative (FN)	True negative (TN)

## Methods

In this study, we propose a novel GP-Net framework to recognize AD from normal controls, which is indeed a binary classification problem in NLP.

### Feature Purification Network: GP-Net

**Figure 1:**
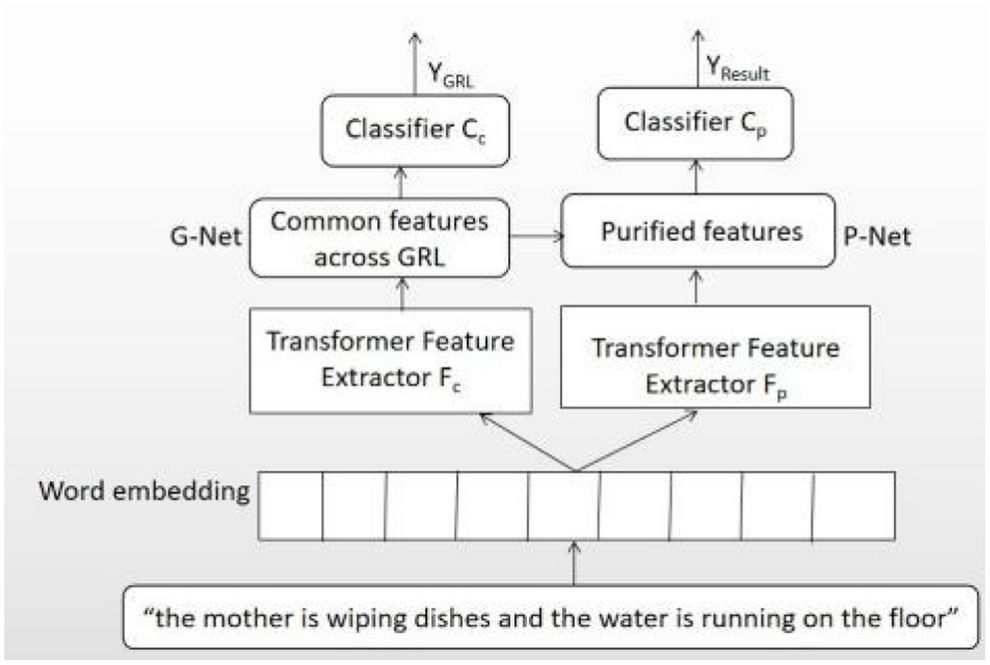
The architecture of GP-Net.

This paper proposed a novel architecture, named GP-Net, to recognize AD, the network structure of which is shown in Figure 1. The whole network includes two sections: G-Net and P-Net. The aim of G-Net is to extract common features by reversing the gradient direction in the training process, and these common features are shared by both classes and have no discriminative for classification. The aim of P-Net is to purify the features further by deleting the common features from traditional features extracted from transformer model. G-Net includes four sections, that is, the input layer X, feature extractor F_c_, GRL, and classifier layer C_c_. P-Net also includes four sections, which include the input layer X, feature extractor F_p_ (the features extracted by F_c_ and F_p_ have no share parameters), purification network, and classifier layer C_p_. The main idea of proposed network is as follows: the feature vector, extracted by the feature extractor F_p_, deletes the common features got from G-Net, and then, more discriminative purified features have got for the final classification. Two operations, including G-Net and P-Net, are required in order for feature purification operation.

### Transformer Extractor

This study uses transformer encoder as the feature extractor. Transformer is a SOTA model which has a novel architecture to solve sequence to sequence tasks. The model can capture long-distance dependencies and learn global semantic features of input text thoroughly through multihead self-attention mechanism. As transformer has some mechanisms as self-attention and location code, it has excellent feature extraction and semantic abstract competence. Like most Seq2Seq model, transformer model also uses encoder–decoder structure, the encoder of which is a better feature extractor with multihead attention and feed forward neural network.

Supposing G-Net and P-Net have the same input *X*, the feature extractors of G-Net and P-Net are F_c_ and F_p_, which can get the advanced features f_p_ and f_c_ from the input layer, respectively, but there are not any shared parameters between them. We refer to the features of P-Net and G-Net, respectively, as


(1) $$\begin{array}{l} f_{p}=Transformer_{p}\left(X\right), \end{array}$$

(2) $$\begin{array}{l} f_{c}=Transformer_{c}\left(X\right), \end{array}$$

Additional details of G-Net and P-Net will be introduced in G-Net and P-Net module.

### G-Net Module

The main goal of G-Net module is to extract common features among datasets, which is not discriminative for the classification. As common features are those shared by all the classes, the classifier cannot use them to distinguish different classes effectively. To get common features, GRL (12, 13, 18) is added between the feature extractor F_c_ and the classifier to reverse the gradient direction. The common features that are shared among different classes are obtained after the training module. G_λ_ can be thought as two incompatible equations that describe the forward and back propagation behaviors:


(3) $$\begin{array}{l} G_{\lambda }\left(x\right)=x, \end{array}$$

(4) $$\begin{array}{l} \frac{\partial G_{\lambda }}{\partial x}=-\lambda I, \end{array}$$

where λ is a hyper parameter. We process feature vector f_c_ through GRL and get f_c_', for example, G_λ_ (f_c_) = f_c_'. To make f_c_' close to real common features, GRL acts as identity transform during the forward propagation and then takes the gradient from subsequent level and changes the value (i.e., multiplies it by -λ) before passing it to the next layer during back propagation, and this operation can ensure that the feature distributions are similar and as indistinguishable as possible for the classifier. Only in this way we can get the common features sharing among classes. Finally, f_c_' is fed to classifier C_c_.


(5) $$\begin{array}{l} Y_{GRL}=softmax\left(W_{c}^{*}f_{c}^{\prime }+b_{c}\right), \end{array}$$

(6) $$\begin{array}{l} Loss_{c}=CrossEntropy\left(Y_{True},Y_{GRL}\right), \end{array}$$

where W_c_ and b_c_ are the weight and bias of classifier C_c._ By optimizing Loss_c_, the feature extractor F_c_ can extract common features of different classes.

### P-Net Model

The goal of P-Net is to extract the semantic information from input example first and then purify features for the classification. Supposing the traditional feature vector we extracted by transformer is f_p_, the common feature vector is f_c_. The final feature vector for classification is f_w_.


(7) $$\begin{array}{l} f_{w}=f_{p}-f_{c}, \end{array}$$

As f_c_ disturbs the classification result, we delete f_c_ from f_p_ to eliminate the influence of nondiscriminative feature vector (i.e., common features), so the feature vector f_w_ is more discriminative than f_p_. Finally, the purification feature vector f_w_ is fed to classifier C_p_.


(8) $$\begin{array}{l} Y_{Result}=soft\;max\left(W_{p}*f_{w}+b_{p}\right), \end{array}$$

(9) $$\begin{array}{l} Loss_{p}=CrossEntropy\left(Y_{True},Y_{Result}\right), \end{array}$$

where W_p_ and b_p_ are the weight and bias of classifier C_p_. By optimizing Loss_p_, the feature extractor F_p_ can purify the features, Loss_c_ and Loss_p_ are trained simultaneously, but they use different optimizers. Loss_c_ use moment SGD optimizer because Ganin and Lempitsky (13) also used moment SGD, and Loss_p_ use Adam optimizer. We also conducted the experiments using Adam optimizer for both G-Net and P-Net and found that the results made no difference when using two different optimizers. In terms of optimization targets of feature extractor F_c_, though the two losses are opposite to each other, a balance can be found to make the extracted feature f_c_ closer to real common features. The algorithm description of the whole training process is shown in Algorithm 1:

**Algorithm 1:** GP-Net

1: Input: Supposing the datasets are $D={\left\{\left(x_{i},y_{i}\right)\right\}}_{i=1}^{N}$, x_i_ is the embedding matrix of deep learning, $X_{i}\in R^{Lk}$, y_i_ is the corresponding classes; randomly initialized the parameters of GP-Net. 2: **For** every iteration b=1, 2,...N, **do**: 3: Sample one batch x_b_ from D, 4: **G-Net section:** 5: Generate common features (CFs) (Equation 1) 6: CFs go through GRL and get the features closer to the common features (Equation 3) 7: Do the classification (Equation 5) 8: **P-Net section:** 9: Generate traditional features(tfs) (Equation 2) 10: Get purified features (Equation 7) 11: Do the classification (Equation 8) 12: **Update parameters:** 13: The parameters of P-Net and G-Net are updated together (Equation 6 and Equation 9) 14: **End for**

## Experiment

### Datasets

Three datasets are used to carry out the experiments which are all the dialogues of picture description task, including English and Chinese.

#### Pitt Datasets

This is a Pitt corpus (43) from Dementiabank dataset (43), which comes from a study at School of Medicine in Pittsburgh University and is gathered longitudinally every year. More detailed description about the dataset can refer to the study (43). After deleting some unqualified datasets such as unknown label, memory impairment, and other dementia diagnose, for example, vascular dementia, there are 498 participants enrolled in our study after data preprocessing, which is composed of 242 controls and 256 possible or probable AD. Both categories are balanced basically.

#### ADReSS Datasets

The datasets include 78 dementia patients and 78 normal controls from ADReSS challenge in 2020. The speech is segmented using a voice activity detect method based on signal energy value. All datasets have already been preprocessed by removing noise.

#### iFLY Datasets

The Chinese datasets include 111 CTRL and 68 AD, with 60 women and 51 men in CTRL group and 38 women and 30 men in AD group, respectively. More details can refer to the website: http://challenge.xfyun.cn/2019/gamedetail?blockId=978.

### Feature Parameters

In the training stage of GP-Net module, a stochastic gradient of 0.9 is used as momentum, and annealing learning rate can be calculated by the following formula:


(10) $$\begin{array}{l} l_{p}=\frac{l_{0}}{{\left(1+\alpha^{*}p\right)}^{\beta }} \end{array}$$

where *l*_0_= 0.01, α= 10, β=0.75, *p* is training progress linearly changing from 0 to 1. In Equation (4), the parameter λ is set as [0.05, 0.1, 0.2, 0.4, 0.8, and 1.0]. Transformer encoder is used as the feature extractor, with three blocks and single head specifically.

### Experiment Results

For our model, 5-fold cross validation was used for training dataset. The dataset was divided into five parts randomly, of which four parts were used for training, and one part was used for test. We repeat the process five times using different test dataset every time. Finally, the results of five times were summarized, and the average value was used as the estimation of model performance index. The classification of our model adopts the following indexes: accuracy, precision, recall, and F1 score are used as the final index (44). The relationship between the actual class and predicted class is shown in Table 1, and the evaluate metrics in this study are defined as Equations (11–14).


(11) $$\begin{array}{l} Accuracy=\frac{TN+TP}{TN+FP+FN+TP} \end{array}$$

(12) $$\begin{array}{l} \text{Precision}=\frac{TP}{TP+FP} \end{array}$$

(13) $$\begin{array}{l} \text{Recall Rate}=\frac{TP}{TP+FN} \end{array}$$

(14) $$\begin{array}{l} F1-score=\frac{2TP}{2TP+FP+FN} \end{array}$$

Table 2 is the classification scores for AD and CTRL on Pitt Dementiabank datasets, including handcrafted features extracted methods and deep learning methods. As far as we know, SOTA on Pitt corpus is the study of Roshanzamir et al. (48) in 2021, and our method in this paper performs better than SOTA. Also, transformer+FP^25^ is the feature project method for text classification, and we did the experiment with this method on our Pitt datasets; the performance of our method is better than the project method with the same datasets. To further compare with some proposed popular pretrained models in recent years, including Bert (37), ERNIE (38), RCNN (31), and DPCNN (32), we do the comparative experiments with the combination models, including BertRCNN, BertDPCNN, BertLogistic, and ERNIEDPCNN models, which are the combination of Bert + CNN, Bert + RCNN, Bert + DPCNN, Bert + Logistic Regression, and ERNIE + DPCNN, respectively. The former is the feature extractor and the latter is the classifier. The evaluation index is shown in Table 3.

**Table 2:** AD vs. CTRL classification scores on Pitt datasets.

**Method**	**Embedding**	**Classifier**	**Precision**	**Recall**	**Accuracy**	**F1**
Sweta Karlekar (23)	POS	CNN-RNN	-	-	91.1	-
Fritsch et al. (29)	n-gram	NNLM+LSTM	-	-	85.6	-
Orimaye et al. (41)	n-grams	D2NN	-	-	88.9	-
Fraser et al. (43)	35 Hand-Crafted Feature	LR	-	-	81.92	-
Yancheva et al. (45)	12 Cluster-Based Features+LS&A	Random Forest	80.00	80.00	80.00	80.00
Sirts et al. (46)	Cluster+PID+SID Features	LR	74.4 ± 1.5	72.5 ± 1.2	-	72.7 ± 1.2
Hernandez et al. (47)	105 Hand-Crafted Features	SVM	81.00	81.00	79.00	81.00
Roshanzamir et al. (48)	BERT _Base_ (Sentence Level)	LR	90.31 ± 7.36	76.52 ± 8.06	84.46 ± 6.31	82.72 ± 7.21
Roshanzamir et al. (48)	Bert _Large_	LR	90.57 ± 3.18	84.34 ± 7.58	88.08 ± 4.48	87.23 ± 5.20
Pan et al. (49)	GloVe Word Embedding Sequence	BiLSTM|GRU Hierarchical Attention	84.02	84.97	-	84.43
Li et al. (50)	185Hand-Craft Features	LR	-	-	77	-
Fraser et al. (51)	Info and LM Features	SVM	-	-	75	77
Transformer+FP^25^	Transformer +Feature projection	Transformer	88	**91**	90.3	90.6
Transformer+GP	Transformer+Feature purification	Transformer	**94**	89	**93.5**	**91.19**

*The study marked with bold is the best performances on Pitt dataset*.

**Table 3:** The result of pre-trained models on Pitt dataset.

**Model**	**Embedding**	**Classifier**	**Precision**	**Recall**	**Accuracy**	**F1**
BertCNN	Bert	CNN	58.85	56.25	56.25	52.79
BertRCNN	Bert	RCNN	-	50.00	50.00	33.33
BertDPCNN	Bert	DPCNN	41.11	47.92	47.92	35.59
ERNIEDPCNN	ERNIE	DPCNN	-	50.00	50.00	33.33
BertLogistic	Bert	Logistic Regression	88	85	86.20	85.60
Transformer+GP	Transformer	Transformer	94.00	89.00	93.50	91.19

From Table 3, we can find that the performance of first four models is not better, with an accuracy of only 50% or so, BertLogistic model has a better accuracy of 86.2%, and our method gets the best result than these pretrained models. In the meanwhile, to prove the superior of our method, we also test the method on ADReSS and iFLY datasets. The result is shown in Table 4, accuracy is improved by 2.1, 4.3, and 2.1%, respectively, on Pitt, ADReSS, and iFLY dataset, which means that the purified features are more discriminative than the features extracted by transformer encoder. Though we do not get SOTA accuracy on ADReSS and iFLY dataset, the performance of our method is improved than transformer.

**Table 4:** Accuracy on three datasets.

**Model**	**Pitt**	**ADReSS**	**iFLY**
Transformer	91.4	74.3	81.6
Transformer+GP	93.5	78.6	83.7

## Discussion

Why the performance is better after purification? We know that transformer is superior to RNN, CNN on its long-distance reasoning ability, but it is not easy to understand the deep semantic feature vector extracted by transformer as deep learning is a “black-box.” The common features in the study are the vector that cannot differentiate for classification in semantic space. They may be the words or sentences that are unimportant, unmeaningful, and irrelevant that may disturb the final classification. Our original dataset is a dialogue of description. It should include some important people, scenes, and ongoing events in the picture. The study (52) pointed out the seed words of the picture should include the following 23 words: boy, girl, woman, cookie, stool, sink, overflow, fall, window, curtain, plate, cloth, jar, water, cupboard, dish, kitchen, garden, take, wash, reach, attention, and see. The sentences including these words are helpful for the classification. Other unrelated words or sentences such as “Can you tell me”, “look, there is no people outside” which are unhelpful words or sentences that cannot distinguish cognitive condition. They are, which we think maybe the common features, unhelpful and may even disturb the final classification. When we rule out these words or sentences (i.e., common features) that disturb the classification, the result can be improved correspondingly. The features extracted manually in this area usually include part-of-speech (POS), fluency, semantic feature, lexical richness, and so on. Now, there is an opinion that the features that deep learning extracted automatically maybe are much like the features that people extract manually, and deleting those unhelpful words for the classification can improve the classification performance.

We know that in transformer model, complexity per layer of self-attention is O(n^2^^*^ d), where d is the representation dimension, and n is the sequence length. Our model includes two sections, one is transformer encoder, the other is feature purification layer which just multiply (-λ) when running back propagation. Both of them can run concurrently and have the same complexity, so the computational complexity of our model is the same as that of self-attention.

## Conclusions

Nowadays, many medical problems used artificial intelligence method to solve (53, 54). which is low cost and convenient. Two methods, that is, feature extraction manually and automatically by deep learning, are usually used to recognize disease. Features extracted method manually based on machine learning does not generalize well, as it needs many special knowledge and annotation to extract features. Due to high cost of manual annotation, it is not feasible to procure numbers of annotated datasets for most clinical tasks. But deep learning does not need any annotation and can finish the process automatically. This paper combines transformer-based model with a feature purification network to improve the classification performance to a large extent. We pretrain transformer and then fine-tune the model on new datasets to transfer learned knowledge to our text classification task. Our work is obviously different from the former studies in AD recognition because none of the former studies improve representation learning of deep learning in this area, as far as we know. The common features extracted by GRL maybe the words that shared by different classifications, or nonimportant words that have small role for classification, ruling out them from traditional representation vector can improve the performance of the model. In addition, we can develop WeChat procedure or APP in mobile device further in order that the elderly can test their cognitive condition at home. So, large volumes of patient's datasets need to be transferred to central cloud server for data analysis, the safety of which is important, and blockchain technology is a better choice which may ensure the security of medical data (53, 55).

Transformer model is still the most widely used deep learning algorithm, but the time complexity of self-attention is higher, which hinders the development of the model, so the improvement of model efficiency is of great importance in the future. Transformer, as the feature extractor we used in this study, can also be replaced by other deep learning algorithms such as Bert, RNN, CNN, and so on; next, we will perfect the work further. In the meanwhile, we also believe that our feature purification method may predict other diseases that language and cognitive impairment related, such as Parkinson's disease, Aphasia, and Autism spectrum disorder. Aphasia is maybe more pronounced as Aphasia is a disease of the brain tissue associated with language function. Our method provides a feasible solution for detecting patients with AD at the doorsteps. Feature purification method for deep learning, as far as we think, is a promising direction to explore in the future.

## Data Availability Statement

The public datasets we used can get from the website: https://sla.talkbank.org/TBB/dementia/English/Pitt, or visit our github website: https://github.com/lzy1012/Public-Pitt-Dementiabank-Dataset.

## Author Contributions

ZY designed the research. QT analyzed the data and interpreted the analysis. NL and ZY wrote the main manuscript text and revised carefully. All authors reviewed and approved the final manuscript.

## Funding

This research was funded by grants of Natural Science Foundation of Zhejiang Province (LGF20F020009), Anhui Provincial Natural Science Foundation (No. 2108085QF269), and the 4th Graduate Student Innovation and Entrepreneurship Competition of Hangzhou Normal University.

## Conflict of Interest

The authors declare that the research was conducted in the absence of any commercial or financial relationships that could be construed as a potential conflict of interest.

## Publisher's Note

All claims expressed in this article are solely those of the authors and do not necessarily represent those of their affiliated organizations, or those of the publisher, the editors and the reviewers. Any product that may be evaluated in this article, or claim that may be made by its manufacturer, is not guaranteed or endorsed by the publisher.
